# Exploring health literacy among Japanese and international university students in Japan: A comparative cross-sectional study

**DOI:** 10.1016/j.jmh.2025.100334

**Published:** 2025-04-25

**Authors:** Akindele Abimibayo Adeoya

**Affiliations:** Medicine and Science in Sports and Exercise Laboratory, Tohoku University Graduate School of Medicine, Sendai, Miyagi, Japan; Division for Interdisciplinary Advanced Research and Education, Tohoku University Advanced Graduate School, Sendai, Miyagi, Japan

**Keywords:** Health literacy, international students, japanese students, university students, health education, health promotion, disease prevention

## Abstract

**Objective:**

Health literacy (HL) is the ability to access, understand, evaluate, and apply health information for well-being. However, comparisons between domestic and international students remain limited. This study aims to investigate HL among Japanese and international university students in Japan and explore the factors that influence it.

**Methods:**

This cross-sectional study used both the English and Japanese versions of the 47-item European Health Literacy Survey Questionnaire (HLS-EU-Q47). Using convenience sampling, a total of 1366 university students across six regions in Japan who provided informed consent participated in this self-administered, online-based survey. Descriptive statistics, *t*-tests, ANOVA and multiple regression were conducted as appropriate at a 0.05 alpha level using JMP statistical software (version 17.0.0)

**Results:**

The results revealed that 60 % and 32 % of participants had inadequate and problematic HL, respectively, indicating that 92 % of all students had limited HL. International students exhibited better HL than Japanese university students (*p* < 0.0001), a difference that remained after adjusting for sociodemographic and educational factors (β = 3.39, 95 % confidence interval = 2.83 – 3.95, *p* < 0.0001). The competency of “appraising” within the healthcare domain presented the greatest challenge for international students, whereas “understanding” within the disease prevention domain was most difficult for Japanese students. Furthermore, the results indicated a strong association between HL and sociodemographic factors such as age, level of study, marital status, and religious affiliation. In contrast, health literacy showed an inverse association with economic status, program of study and parental education level. There was an observable trend between improved Japanese language proficiency and improved HL among international students.

**Conclusion:**

International students in Japan demonstrated better HL than Japanese university students. Educational institutions must take a more proactive role in fostering HL for all students through general health education and peer-to-peer programs to create a more informed, healthy, and productive student community.

## Introduction

Health literacy (HL) is crucial in addressing the challenges posed by preventable diseases, increasing mortality rates, and soaring medical costs. HL can be defined as the ability to “access, understand, appraise, and apply health information for decision-making in healthcare, disease prevention, and health promotion to maintain and improve quality of life (QoL) throughout an individual’s lifespan” (Sørensen et al., 2012). It integrates personal capabilities with the demands of the healthcare system, culture, and social capital (Nutbeam, 2008). As a vital determinant of health, HL bridges the gap between quality healthcare, service utilization, health disparities, and enhancements in QoL (Nakayama, 2014; Nutbeam and Lloyd, 2021).

The dimensions of HL encompass three domains: healthcare, disease prevention, and health promotion. Each domain involves four competencies: the abilities to access, understand, process, and apply relevant health information (Sørensen et al., 2012; Sørensen et al., 2015). The healthcare domain involves seeking, interpreting, and using medical information to make informed decisions and follow medical advice. Disease prevention focuses on identifying and evaluating risk factors to make preventive health decisions. Health promotion emphasizes staying informed about health determinants in the social and physical environment and making decisions to enhance well-being. Each competency plays an essential role in enabling individuals to navigate health-related decisions and maintain overall well-being (Sørensen et al., 2012; Sørensen et al., 2015). HL extends beyond reading and understanding prescriptions; it is influenced by an individual’s circumstances, environmental factors, needs, and the complexity of the healthcare system (Parnell et al., 2019). (Sørensen et al., 2013) describe HL as a result of both informal and formal learning and health education, which are dynamic and evolve over time. HL is multidimensional and encompasses culture, context, and language (Sørensen et al., 2012; Nutbeam, 2008; Baker, 2006; Sørensen, 2019), establishing itself as a critical component of health promotion.

The challenges of HL are multifaceted. Limited HL, which includes both inadequate and problematic levels, is associated with adverse outcomes such as poorer health outcomes and poorer use of health care services (Berkman et al., 2011), reduced adoption of protective behaviors such as immunization, and an inadequate understanding of antibiotics (Castro-Sánchez et al., 2016), risky behaviors such as smoking (Li et al., 2022). Global studies reveal a widespread epidemic of limited HL among the general population (Sørensen et al., 2015; Nakayama et al., 2015; Centers for Disease Control and Prevention (CDC) 2023; Rootman and Gordon-El-Bihbety, 2008; Liu et al., 2018; Rajah et al., 2019; McClintock et al., 2020; Mávita-Corral, 2018; Australian Bureau of Statistics 2006; Satherley and Lawes, 2008; Özkan et al., 2021; Hoffman et al., 2016; Paiva et al., 2017; Chan et al., 2015), with subtantial implications for the economic efficiency of healthcare systems and societal well-being. Limited HL among university students (Kühn et al., 2022), similar to that in the general population, presents a unique concern, given their role in shaping the future. In Japan, for instance, HL levels among the general population (Nakayama et al., 2015) are comparable to those of university students (Yokoyama et al., 2023). Since high HL has been linked to improved subjective health in this population (Yokoyama et al., 2023), the high prevalence of limited HL is particularly worrisome.

University students represent a distinct population facing numerous challenges, including academic demands, economic pressure, social identity development, adjustment to living independently, future aspirations, and life post-graduation uncertainty. These challenges can adversely affect physical and mental health, sleep patterns, health behavior, QoL (Ribeiro et al., 2018; Pascoe et al., 2020), and even mortality. Students often experience higher levels of psychological distress than non-students (Stallman, 2010). This situation might be more dire for international students who, in their quest for education, leave their familiar social environments and must navigate the added complexities of adjusting to a new culture and their status as immigrants. Numerous studies have examined HL among the general population; however, gaps remain in understanding this phenomenon among university students, particularly how HL differs between domestic and international students. This gap was highlighted in a systematic review by Kühn et al. (Kühn et al., 2022). Additionally, in Japan, there is a lack of comprehensive studies using geographically representative samples of students. Therefore, this study’s primary objective is to conduct a comparative assessment of HL among Japanese and international university students in Japan, specifically investigating how sociodemographic characteristics influence students’ HL, their competencies across the three domains of HL, as measured by the HLS-EU-Q tool, and the impact of Japanese language proficiency and length of stay in Japan on international students’ HL.

## Materials and methods

### Study design and participants

This study is part of the larger J-IMPACT Study (Japan International Migrant Population and Comparative Health Study), a nationwide case-control study that evaluates various health indices among migrants and the domestic population in Japan. Findings from other parts of this research will be reported separately. This cross-sectional study surveyed both Japanese and international students at universities in Japan. The eligibility criterion was being a currently enrolled student in Japanese universities. Data collection occurred from February 20 to August 10, 2023. Before participating in the web-based survey, designed using Google forms, a cloud-based software, students provided informed consent on the survey’s first page. This included comprehensive information about the study’s purpose, voluntariness, confidentiality, and the ethical approval obtained from the Tohoku University Research and Ethics Committee (approval number: 2022–1-921) prior to the commencement of the study.

### Sampling technique

To achieve a substantial response rate and geographical representation, purposive sampling was employed to select seven universities from the 30 Japanese universities hosting the highest number of international students (Japan Student Service Organization (JASSO) 2022). These universities are located across six of Japan’s eight regions: Kanto, Kinki, Kyushu, Tohoku, Hokkaido, and Chubu. To ensure participation of only eligible students, the online survey link (QR code) was self-administered by the researcher and/or research assistants at each selected university using convenience sampling method. A total of 1366 respondents completed the survey.

### Instruments and procedure

The instruments were available in both English and Japanese versions, allowing participants to choose their preferred language option.

#### Health literacy

The 47-item version of the HLS-EU (HLS-EU-Q47) was utilized to measure HL (Sørensen et al., 2015; Sørensen et al., 2013). This standardized instrument, validated and widely used among the general population in Asia (Duong et al., 2017), including Japan (Nakayama et al., 2015), and among university students (Yokoyama et al., 2023; Sukys et al., 2017), rates the perceived difficulty of each item on a 4-point Likert scale (1=very difficult, 2=difficult, 3=easy, 4=very easy). A comprehensive HL index was constructed using scores from all items, standardized to a unified metric ranging from 0 to 50 using the formula: (MEAN–1) × (50/3), following the method established by the developer (Sørensen et al., 2013). Responses marked “do not know” were treated as missing values as also described by previous studies (Nakayama et al., 2015; Ishikawa and Kiuchi, 2019; Rueda-Medina et al., 2020). MEAN represents the average of all item responses for each participant. HL index scores were calculated for respondents providing valid answers for at least 80 % of the items across all indices for general HL (GEN—HL) (*n* = 1307), healthcare HL (HC—HL) (*n* = 1300), disease prevention HL (DP-HL) (*n* = 1310), and health promotion HL (HP-HL) (*n* = 1321), as well as the four HL competencies: accessing (*n* = 1273), understanding (*n* = 1333), appraising (*n* = 1285), and applying (*n* = 1314). Data from approximately 96 % of respondents were included in the analysis. In line with previously validated studies, HL levels were categorized into four groups: inadequate (0–25), problematic (>25–33), sufficient (>33–42), and excellent (>42–50), with limited HL defined as the combined total of inadequate and problematic levels (Sørensen et al., 2015; Sørensen et al., 2013; Nakayama et al., 2015). The Cronbach’s alpha coefficients for the English, Japanese, and combined versions of the scale are 0.94, 0.94, and 0.94, respectively.

#### Demographic characteristics

Sociodemographic and cultural indices included nationality (Japanese, International), gender (male, female, others) and age categories (<20 years, 20–24 years, 25–30 years, and ≥31 years). The location of universities by prefecture was dichotomized into megacities (Tokyo and Osaka) and other metropolitan cities (Fukuoka, Ibaraki, Miyagi, Hokkaido, and Aichi). Fields of study were divided into medical sciences and non-medical programs. Study levels were categorized into undergraduate and postgraduate, and marital status into single or married. Religious adherence was dichotomized into adherence and no religion. Parents’ highest level of education was categorized from no education/compulsory education to university degree or higher. Economic status was self-rated and ranked on a 10-point scale, ranging from 1 (lowest) to 10 (highest). For analysis, these were categorized as low (1–4 points), moderate (5–7 points), or high (8–10 points). Japanese language ability was self-reported on a five-point scale: cannot speak, can speak a little Japanese, able to communicate about daily life, able to report and understand information using medical terms, or speaks it fluently (Soneta et al., 2021). Length of stay in Japan was categorized as ≤1 year, 1–2 years, 3–4 years, or 5 years or more.

#### Data analysis

All data were analyzed using John’s Macintosh Project version 17.0.0 (Statistical Discovery LLC, Cary, NC, USA) employing both descriptive and inferential statistical methods. Descriptive statistics provided an overview of the distribution of HL indices and compared these indices across different groups. For inferential analysis, *t*-tests compared HL indices between Japanese and international students. A one-way ANOVA and *t*-tests were used as appropriate to examine the influence of sociodemographic characteristics on HL. To examine the independent association between nationality and HL, multiple linear regression analyses were conducted, adjusting for potential sociodemographic and educational confounders. Model 1 controlled for gender and age, while Model 2 additionally adjusted for level of study, program of study, marital status, parents’ level of education, religion, and economic status. Statistical significance was set at *P* < 0.05.

## Results

The distribution of respondents by nationality included 859 (63 %) Japanese and 507 (37 %) international students.

### Health literacy among university students in Japan

Table 1 details the HL indices of university students in Japan. Analysis of GEN—HL revealed a mean score of 22.9 (SD=8.0) for the 1307 valid responses. A majority (60 %) were categorized as having inadequate HL, followed by 32 % with problematic HL, 7 % with sufficient HL, and only 1 % exhibited excellent HL. Overall, 92 % were classified as having limited HL, defined as the combined total of inadequate and problematic levels. Similar patterns were observed in the HC, DP, and HP domains, underscoring the prevalence of limited HL among university students.

**Table 1:** Health literacy index of Japanese and international university students in Japan.

Health literacy	Total				Japanese					International					
	N	HL levels %	M	SD	n	HL levels %	M	SD	95 %CI	n	HL levels %	M	SD	95 %CI	P^a^
**GEN—HL**	1307		22.9	8.0	847		20.6	7.4	20.13–21.13	460		27.2	7.4	26.58–27.95	<0.0001
Inadequate		60				73.2					36				
Problematic		32				23.5					47				
Sufficient		7				3.2					15				
Excellent		1				0.1					2				
Limited*		92				96.7					83				
**HC—HL**	1300		22.8	8.5	843		21.0	8.3	20.51–21.64	457		26.1	7.8	25.47–26.91	<0.0001
**DP-HL**	1310		21.9	9.6	848		18.5	8.1	17.95–19.05	462		28.2	8.9	27.41–29.04	<0.0001
**HP-HL**	1321		23.9	9.6	848		22.1	8.6	21.53–22.70	473		27.2	8.9	26.43–28.04	<0.0001

⁎ Limited (Inadequate+problematic).

a t-test.

Table 2 presents the HL competence of university students in Japan. In this assessment, “understanding” emerged as the most challenging area, with a mean score of 19.4 (SD=10.1). Conversely, “appraising” showed relatively better outcomes, with a mean score of 24.9 (SD=8.9). Nonetheless, across all competences, a significant proportion of students displayed limited HL.

**Table 2:** Health literacy competence of Japanese and international university students in Japan.

Competence Domain	Total			Japanese				International				
	N	M	SD	n	M	SD	95 %CI	n	M	SD	95 %CI	P^a^
**Accessing**	1273	22.4	8.7	830	20.3	8.2	19.81–20.94	443	26.2	8.3	25.47–27.03	<0.0001
**Understanding**	1333	19.4	10.1	852	15.3	8.1	14.83–15.92	481	26.7	9.1	25.97–27.61	<0.0001
**Appraising**	1285	24.9	8.9	841	24.8	9.3	24.21–25.47	444	25.2	8.1	24.46–25.99	0.46
**Applying**	1314	22.4	8.6	850	20.0	8.0	19.47–20.56	464	26.8	8.0	26.07–27.53	<0.0001

a t-test.

### Health literacy comparison between Japanese and international university students in Japan

Table 1 shows a comparison of the HL indices between Japanese and international students, revealing significant disparities across all HL domains. International students consistently had higher levels of HL (*P* < 0.0001). For instance, in the GEN—HL domain, the mean score for international students was 27.2 (SD=7.4), notably higher than the 20.6 (SD=7.4) for Japanese students. This difference was also reflected in the percentages of limited HL, comprising 83 % of international students and 96.7 % of Japanese students.

Table 2 presents an analysis of HL competence by nationality. International students had higher mean scores than Japanese students across all competences (*P* < 0.0001). Notably, in the “understanding” competency, international students achieved a mean score of 26.7, higher than the 15.3 observed among Japanese students. Table 3 presents further analysis focused on domain-specific competences indicated that international students demonstrated higher levels of HL competences across most domains. However, an exception was noted in the “appraising” competence within the healthcare domain, where Japanese students showed a higher mean score of 28.5 compared to the 23.7 score of their international counterparts (*P* < 0.0001).

**Table 3:** Comparison of health literacy competence by domain between Japanese and international university students in Japan.

Domain	Competence	Total			Japanese			International			*t*-test	
		N	M	SD	n	M	SD	n	M	SD	t	P
Healthcare	Accessing	1264	24.5	10.1	813	23.1	10.2	451	27.1	9.4	6.89	<0.0001
	Understanding	1270	20.5	11.0	830	18.2	10.7	440	24.9	10.1	10.79	<0.0001
	Appraising	1196	26.9	10.8	789	28.5	10.9	407	23.7	9.8	−7.34	<0.0001
	Applying	1222	19.0	11.5	817	14.3	9.3	405	28.5	9.6	24.82	<0.0001
Disease Prevention	Accessing	1255	22.1	11.2	820	18.6	10.0	435	28.5	10.3	16.30	<0.0001
	Understanding	1316	16.7	15.8	846	8.7	10.2	470	31.3	13.5	34.17	<0.0001
	Appraising	1318	24.2	10.3	847	23.3	10.6	471	25.7	9.7	3.96	<0.0001
	Applying	1277	22.3	11.1	830	19.6	10.5	447	27.5	10.3	12.91	<0.0001
Health Promotion	Accessing	1300	21.7	10.3	840	20.1	9.9	460	24.8	10.2	8.08	<0.0001
	Understanding	1308	21.3	11.2	845	17.8	10.1	463	27.6	10.4	16.45	<0.0001
	Appraising	1303	25.6	12.1	827	23.8	12.3	476	28.5	11.0	6.85	<0.0001
	Applying	1295	26.9	11.7	838	26.7	11.9	457	27.2	11.3	0.70	0.48

### Association between sociodemographic factors and HL among university students in Japan

Table 4 presents the association between sociodemographic factors and HL among university students in Japan. Higher HL is associated with increased age (*P* < 0.0001), being a postgraduate (*P* < 0.0001), married (*P* = 0.0002), and a religion adherent (*P* < 0.0001). An inverse association was found between economic status (*P* = 0.001), program of study (*P* < 0.0001) and parents’ education (*P* = 0.0008). No association was found for gender (*P* = 0.97) and city type (*P* = 0.19).

**Table 4:** Association between sociodemographic factors and health literacy among university students.

Variable	GEN—HL (1307)			
	n	M	SD	P
**Gender** Male Female Others	704 582 21	22.9 22.9 22.5	8.2 7.9 8.1	0.97^a^
**Age-group (years)** < 20 20–24 25–30 ≥31	298 680 224 105	21.5 22.2 25.9 25.6	7.5 7.8 7.7 9.3	<0.0001^a^
**Level of study** Undergraduate Postgraduate	854 453	21.8 25.1	7.7 8.2	<0.0001^b^
**Program of study** Medical Non-medical	288 1019	20.9 23.5	8.5 7.8	<0.0001^b^
**Marital status** Single Married	1228 79	22.7 26.2	7.9 9.4	<0.0002^b^
**Economic status** Low Moderate High	202 811 294	23.4 23.4 21.4	8.3 7.6 8.8	<0.0010^a^
**Parents’ level of education** No education/UBE High school Vocational college University	43 157 157 950	27.2 23.5 23.6 22.5	6.4 9.4 7.3 7.9	<0.0008^a^
**Religion** Adherent No religion	482 825	24.0 22.3	8.1 7.9	<0.0001^b^
**City type** Metropolis Mega cities Missing	967 324 16	22.7 23.4	8.1 7.9	0.19^b^

a ANOVA.

b t-test.

### Association between nationality and HL among university students in Japan

Table 5 presents the multiple regression analysis on the association between nationality and HL among university students in Japan. The unadjusted model showed a positive association between being an international student and higher HL compared to Japanese students (β = 3.31, SE = 0.21, 95 % CI = 2.89 – 3.74, *P* < 0.0001). This association remains robust even after controlling for sociodemographic and educational factors in model 2 (β = 3.39, SE = 0.28, 95 % CI = 2.83 – 3.95, *P* < 0.0001), with international students consistently demonstrating higher HL than Japanese students. These findings suggest that nationality is strongly associated with HL.

**Table 5:** Multiple regression analysis on the association between nationality and health literacy.

	Nationality			P
	Japanese	International		
		β (SE)	95 % CI	
Unadjusted model	Reference	3.31 (0.21)	2.89 – 3.74	<0.0001
Model 1^a^	Reference	3.60 (0.26)	3.07 – 4.13	<0.0001
Model 2^b^	Reference	3.39 (0.28)	2.83 – 3.95	<0.0001
R-squared (Adjusted R-squared) = ^un^^a^^djusted model^ 0.15 (0.15), a0.15 (0.15), b0.16 (0.15)				

CI: confidence interval, β: estimate, SE: standard error.

a Adjusted for gender and age.

b Additionally adjusted for level of study, program of study, marital status, parents’ level of education, religion and economic status.

### Impact of Japanese language proficiency and length of stay in Japan on HL among international students

Table 6 details the impact of Japanese language proficiency and length of stay in Japan on HL among international university students, with 460 and 455 valid responses, respectively. A pattern emerged in HL scores based on language proficiency. International students with no Japanese language skills had a lower mean GEN—HL score of 26.4 (SD=8.09). Whereas those proficient in medical terms and fluent in Japanese had higher mean scores of 28.2 (SD=6.9) and 28.1 (SD=9.1), respectively. However, no differences were found based on language proficiency (*P* = 0.71).

**Table 6:** Impact of Japanese language proficiency and length of stay on international students’ health literacy.

Variable		GEN—HL				
		n	Mean	SD	95 %CI	P^a^
**Japanese Language Proficiency (*N*=460)**	Cannot speak	54	26.4	8.0	24.40–28.40	0.71
	Can speak a little	157	27.3	7.1	26.21–28.55	
	Able to communicate about daily life	170	27.0	6.9	25.89–28.14	
	Able to report and understand information using medical terms	25	28.2	6.9	25.31–31.18	
	Speaks fluently	54	28.1	9.1	26.13–30.13	
**Length of stay in Japan** **(*N*=455)**	< 1yr	156	27.9	7.0	26.75–29.09	0.26
	1–2yrs	140	26.8	6.7	25.59–28.06	
	3–4yrs	94	27.9	7.8	26.41–29.42	
	≥5yrs	65	26.1	8.7	24.31–27.93	

^a^ANOVA.

Varied results were revealed according to international students’ different lengths of stay in Japan. Students who had been in Japan for less than a year and 3–4years had a higher and similar GEN—HL score, averaging 27.9. While those who had been in Japan for 5 years or more scored the lower with an average of 26.1 (SD=8.7). However, no differences observed (*P* = 0.26).

## Discussion

The study revealed a high prevalence of limited HL among university students in Japan, at 92 %. This figure is higher than the 85.4 % reported in a sample of the general Japanese population with a similar national geographic representation (Nakayama et al., 2015) and exceeds the 84.6 % reported among university students in Osaka Prefecture (Yokoyama et al., 2023). These findings support (Nakayama et al., 2015) concerns about potentially greater disparities in HL between Japan and Europe than previously reported. They also corroborate the results of a systematic review (Kühn et al., 2022), which found that HL is comparatively low among university students. The HL results of this study exceed those reported in Malaysia (Rootman and Gordon-El-Bihbety, 2008) and align closely with findings from the United States (Centers for Disease Control and Prevention (CDC) 2023); however, they are lower than those observed in the United Kingdom (Simpson et al., 2020; NHS Health Education England 2024). Using the HLS-EU-Q47, the results were lower than the overall figures for Europe (Sørensen et al., 2015). Specifically, this study reported higher levels of limited HL among university students in Japan compared to those in Lithuania (33.1 %) (Sukys et al., 2017), Spain (30.2 %) (Rueda-Medina et al., 2020), Turkey (58.9 %) (Uysal et al., 2020), and Portugal (82.3 % using HLS-EU-Q 16) (Rosário et al., 2024). This research confirms that many individuals struggle to make healthy choices even in countries with robust health systems (The Lancet 2022).

This study revealed that international students possess higher HL levels than their Japanese counterparts. Even after adjusting for sociodemographic and educational factors, international students consistently demonstrated higher HL than Japanese students. These results are consistent with findings from a study at a Hungarian university, which involved 32 international students among undergraduates in health-related fields (Bánfai-Csonka et al., 2022) One contributing factor may be the characteristics of Japan’s healthcare system. Known for its high-quality care, generous coverage at a comparatively low cost, and the freedom for individuals to choose their healthcare facilities without a primary-secondary care distinction, the system is universally accessible (Sakamoto et al., 2018; Aoyama, 2022). However, the absence of a gatekeeper system and the historical lack of a general practitioner system have led to a shortage of certified primary healthcare professionals (Nakayama et al., 2015). This might have resulted in a gap in comprehensive health education. Previous studies have noted the lack of national online platforms providing neutral, authoritative, comprehensive, and understandable health information in Japan, as well as the absence of public schemes for professional health support (Nakayama et al., 2015; Yokoyama et al., 2023). Basic school education in Japan does not adequately address students’ HL (Yokoyama et al., 2023). By contrast, international students often access a broad range of health information sources in both Japanese and their native languages through international communities, academic resources, and language courses, enhancing their HL. While “appraising” competencies particularly in healthcare are challenging for the Japanese general population (Nakayama et al., 2015), they appear to be a strength among Japanese university students.

Japan’s robust institutional safety systems and disaster preparedness, while fostering a sense of security and trust, may inadvertently reduce individual motivation for personal health management and disease prevention. Nakai and Nakano (Nakai and Nakano, 2023) observed, citizens show low self-help practices despite government promotion efforts. This dynamic may also explain the decline in HL among international students over time, as they become accustomed to and reassured by Japan’s safety and disaster preparedness response and management. In addition to the nature of the healthcare system and basic education challenges identified by previous studies (Nakayama et al., 2015; Yokoyama et al., 2023) above, Japanese cultural values like collectivism, respect for authority, and privacy may limit individual initiative in health matters. Coupled with cultural response tendencies, these factors may contribute to lower HL reported among Japanese university students, particularly the differences observed in understanding competencies within the disease prevention domain. According to the World Health Organization, HL is a social practice, shaped by community norms, culture, and broader organizational and political influences that affect health decisions and behaviors (World Health Organization 2022).

The current study corroborates previous findings (Yokoyama et al., 2023) that no gender differences exist in HL among university students in Japan. It also aligns with results among Danish students (Elsborg et al., 2017). However, literature presents divergent results, with some studies reporting higher HL among female students (Sukys et al., 2017; Rosário et al., 2024; Vamos et al., 2016; Rababah et al., 2019), and other indicating higher HL among male students (Budhathoki et al., 2019). Similar disparities are observable in the non-student population, with some studies showing no sex differences (Eronen et al., 2019), some indicating higher HL in women (Nakayama et al., 2015), and others higher HL in men (Liu et al., 2015).

This study also demonstrates that better HL correlates with an increase in age and higher educational level. This finding aligns with previous research on the general population (Nakayama et al., 2015) and university students (Rosário et al., 2024; Vamos et al., 2016; Budhathoki et al., 2019), although (Rababah et al., 2019) report that while the level of study had a detectable effect on HL, age does not. In contrast, (Rosário et al., 2024) reported a positive association with age and an inverse association with the level of study. Being married is associated with improved HL among university students, a factor not previously considered for this demographic (Kühn et al., 2022). Similar findings have been reported for the general population (Eronen et al., 2019; Rikard et al., 2016).

This study unexpectedly revealed that students in health-related fields have lower HL than their counterparts in non-health-related disciplines. This finding aligns with research from China, where (Zhang et al., 2016) reported better HL levels among engineering students than among those studying medical or health-related sciences. However, this insight contrasts with findings from (Rosário et al., 2024; Rababah et al., 2019; Budhathoki et al., 2019) who reported higher HL among university students in health-related disciplines. One possible explanation is the intense specialization in health-related fields, which may limit students’ exposure to a broader spectrum of health issues, confining their knowledge to specific areas. By contrast, students of non-health-related courses often receive a more generalized health education. Self-motivation among students, shaped by personal experiences or individual health status, may also play a role. Furthermore, the results may have been skewed by sample size discrepancies. For instance, students in clinical or experimental medicine may be disproportionately represented in our study compared with those in public health or nursing, who generally receive broader health education and promotion, potentially influencing the observed HL levels. In Denmark, a study (Elsborg et al., 2017) among university students enrolled in health-related study programs (health informatics, medicine, molecular biomedicine, and public health) found that students in the public health program tended to have higher HL scores, whereas those in molecular biomedicine tended to have lower HL scores.

Students from low and moderate economic backgrounds demonstrated significantly higher HL levels than those from high economic backgrounds. However, these findings are inconsistent with those reported by Zou et al. (Zou et al., 2018), who found that Chinese students from average or higher economic backgrounds had higher HL. Meanwhile, students whose parents had no formal education or only basic education demonstrated higher HL, whereas those with university-educated parents showed lower HL. This counterintuitive result might indicate compensatory behavior, where students from less-educated families proactively seek health information. This aligns closely with (Rosário et al., 2024), who found lower health literacy among students with university-educated parents. This contrasts with the positive correlation between parental education levels and HL (Elsborg et al., 2017; Zhang et al., 2016). Notably, Zhang et al. (2016) used only two categories for parental education: “no high school” and “high school attainment.”

Adherents of any religion demonstrated markedly higher HL across all domains than those without a religious affiliation. Religious affiliation is linked to enhanced social support, increased marital stability, a greater sense of purpose in life, and higher levels of happiness and subjective well-being (VanderWeele, 2017), as well as improved health (Koenig, 2012). No differences in HL levels based on city type indicate that urbanization levels may not critically influence HL among university students in Japan. This aligns with findings (Nakayama et al., 2015) among the general Japanese population and (Zou et al., 2018) among university students in urban and rural areas of China.

In Addressing limited HL among university students in Japan, educational institutions should implement tailored interventions, such as general health education, that harmonize treatment-focused knowledge with a strong emphasis on preventive care and health promotion. Encouraging independent health information-seeking, critical assessment, and open discussions through peer-to-peer activities will empower students as informed health consumers and help bridge HL gaps across diverse student demographics. Policies promoting language support services for students with lower Japanese proficiency strategies can enhance HL. Systematically addressing these educational and cultural aspects can improve HL among university students, foster a population capable of making informed health decisions, and achieve optimal health outcomes.

### Strengths and limitations

This study is the first to comparatively examine HL between domestic and international students across disciplines using geographically diverse samples, contributing a novel perspective to the existing literature. Additionally, it incorporates a broader range of sociocultural, economic, and demographic variables than previous research on college students, thereby extending the understanding of HL in the university context and its association with these multifaceted factors. Despite these strengths, the study is not without limitations. The HLS-EU-Q47 instrument, which is primarily based on self-reported data, may not provide an entirely objective assessment of HL. More also, the sampling method and the voluntary nature of participation may introduce selection bias, and the characteristics of respondents limit the generalizability of the findings Furthermore, the study’s cross-sectional design limits the ability to draw direct causal relationships. It is noteworthy that most objective HL scales, such as the Newest Vital Sign and Test of Functional Health Literacy in Adults, are self-reported and often measure competencies in areas such as reading and calculating precise medication dosages. Utilizing these instruments among university participants could potentially lead to a very high rate of correct responses. The HLS-EU-Q47 is widely recognized and used internationally, and both its English and Japanese versions have been validated and verified as reliable. A systematic review comparing performance-based and self-reported HL scales found negligible variances (Kiechle et al., 2015); nevertheless, self-report instruments are subjective evaluation tools.

## Conclusion

This study highlights the higher prevalence of limited HL among university students in Japan compared to the general population. International students in Japan demonstrated significantly better HL than their Japanese counterparts. The study also revealed a strong association between HL and sociodemographic factors, such as age, level of education, marital status, and religious affiliation. Conversely, HL was inversely associated with the economic status, the program of study and the level of parental education. Students in low-moderate economic status, non-health-related fields and those whose parents had lower educational levels tended to have higher HL scores than students in high economic status, health-related fields and those with highly educated parents. There was an observable trend between improved Japanese language proficiency, the level of urbanization (city type), and improved HL. No difference was observed between students’ gender or international students’ length of stay and their HL. These findings highlight the need for educational institutions to play a leading role in fostering HL among students through general health education and peer-to-peer programs/activities. They also emphasize the importance of a comprehensive and inclusive approach to health education in creating a more informed, healthy, and productive student community.

## CRediT authorship contribution statement

**Akindele Abimibayo Adeoya:** Writing – review & editing, Writing – original draft, Visualization, Validation, Methodology, Investigation, Funding acquisition, Formal analysis, Data curation, Conceptualization.

## Declaration of competing interest

The authors declare that they have no known competing financial interests or personal relationships that could have appeared to influence the work reported in this paper.
